# Untargeted mass spectrometry-based metabolomics approach unveils biochemical changes in compound probiotic fermented milk during fermentation

**DOI:** 10.1038/s41538-023-00197-z

**Published:** 2023-05-24

**Authors:** Yaru Sun, Shuai Guo, Ting Wu, Jingwen Zhang, Lai-Yu Kwok, Zhihong Sun, Heping Zhang, Jicheng Wang

**Affiliations:** 1 Key Laboratory of Dairy Biotechnology and Engineering (Inner Mongolia Agricultural University), Ministry of Education, 010018 Hohhot, China; 2 Key Laboratory of Dairy Products Processing, Ministry of Agriculture and Rural Affairs, 010018 Hohhot, China; 3 Inner Mongolia Key Laboratory of Dairy Biotechnology and Engineering, Inner Mongolia Agricultural University, 010018 Hohhot, China

**Keywords:** Symbiosis, Nutrition

## Abstract

Probiotic functional products have drawn wide attention because of their increasing popularity. However, few studies have analyzed probiotic-specific metabolism in the fermentation process. This study applied UPLC-QE-MS-based metabolomics to track changes in the milk metabolomes in the course of fermentation by two probiotic strains, *Lacticaseibacillus paracasei* PC-01 and *Bifidobacterium adolescentis* B8589. We observed substantial changes in the probiotic fermented milk metabolome between 0 and 36 h of fermentation, and the differences between the milk metabolomes at the interim period (36 h and 60 h) and the ripening stage (60 h and 72 h) were less obvious. A number of time point-specific differential metabolites were identified, mainly belonging to organic acids, amino acids, and fatty acids. Nine of the identified differential metabolites are linked to the tricarboxylic acid cycle, glutamate metabolism, and fatty acid metabolism. The contents of pyruvic acid, γ-aminobutyric acid, and capric acid increased at the end of fermentation, which can contribute to the nutritional quality and functional properties of the probiotic fermented milk. This time-course metabolomics study analyzed probiotic-specific fermentative changes in milk, providing detailed information of probiotic metabolism in a milk matrix and the potential beneficial mechanism of probiotic fermented milk.

## Introduction

Fermentation is a metabolic process, in which organic matters are completely decomposed under the action of enzymes^1^. Fermented milk is one of the most important fermented foods, recognized as a healthy food and a good carrier of probiotics^2^. Probiotics are widely distributed in the intestinal tract, oral cavity, female reproductive tract, and even the skin mucosal layer. Probiotic intervention in fermented milk is largely supported by physicians, especially gastroenterologists worldwide^3^. There is growing evidence that probiotic fermented milk confers various health benefits to consumers, such as lowering serum cholesterol, boosting immune responses, improving gut health, preventing various cancers, and mitigating cognitive impairment^2^. These effects can be attributed to various functional components in the probiotic fermented milk, such as peptides, polysaccharides, fatty acids, organic acids, vitamins, and γ-aminobutyric acid (GABA)^4^. *Lacticaseibacillus paracasei* has been used extensively in the probiotic food industry due to its health effects. The health claims are not limited to gastrointestinal health but also host immunity, and recent scientific evidence supports its clinical efficacy on alleviating oral diseases, such as periodontitis and dental caries^5,6^. More importantly, some *Lacticaseibacillus paracasei* strains have good fermentation characteristics and can be used in food fermentation^6,7^. Another group of beneficial bacteria are bifidobacteria. The species *Bifidobacterium adolescentis* has drawn increasing attention because it is an important component of the human gut microbiota that links to host health, such as maintaining a healthy body weight^8^ and preventing constipation^9^. However, *Bifidobacterium adolescentis* is difficult to be used for fermentation due to its low viability^10^. Therefore, compound fermentation of *Bifidobacterium adolescentis* with other strains with good fermentation performance would be one option that increases its viability as probiotics.

Some previous studies have investigated changes in probiotic fermented milk metabolomes, but most studies do not focus on analyzing probiotic-specific function and metabolism in the process^1,11^. *Streptococcus thermophilus* and *Lactobacillus delbrueckii* subsp. *bulgaricus* are the most common basic starter bacteria used in combination with probiotics for producing probiotic milk fermentation^10^. However, the combined use of these traditional starter bacteria with probiotics in fermentation makes it hard to distinguish probiotic-specific biochemical effects and metabolism from those of the traditional starter bacteria. Therefore, it would be of interest to investigate the single effect of probiotics on the milk metabolome, which would provide insights into probiotic-specific metabolism. Moreover, ripening changes in the milk metabolome of probiotic products may occur due to the continuous physiological action of viable microbes even after the fermentation ended. This is of particular importance in probiotic products, as high viability of probiotic bacteria during and after fermentation is crucial for their beneficial effects. However, previous studies usually only analyze the ripening changes without focusing on monitoring metabolite changes during the milk fermentation process, which would also reflect the product quality and stability^11,12^.

**Fig. 1:**
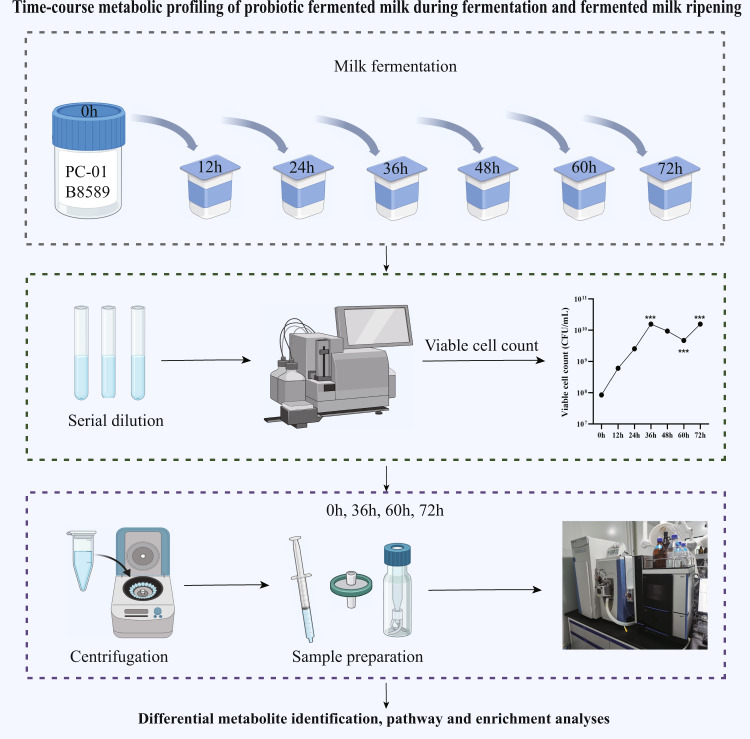
Schematic diagram of study design. Pasteurized milk was co-fermented by two probiotic strains, *Bifidobacterium adolescentis* B8589 and *Lacticaseibacillus paracasei* PC-01. Samples were collected every 12 h of fermentation until the pH reached 3.8 after 72 h. The probiotic viable count in the fermented milk was enumerated by flow cytometry. The milk metabolomes (at 0, 36, 60, and 72 h) were detected by ultra-performance liquid chromatography for tracking changes in metabolites and metabolic pathways of interest in the course of milk fermentation. Finally, enrichment analysis was performed on the identified differential metabolic pathways.

This study applied two probiotic strains in milk fermentation, namely *Lacticaseibacillus paracasei* PC-01 (PC-01) and *Bifidobacterium adolescentis* B8589 (B8589). The PC-01 strain was isolated from naturally fermented yak milk. Because of its good fermentation and probiotics characteristics, it has been successfully applied in fermented milk production^13^. The B8589 strain was isolated from the gut of a healthy infant; and it has good anti-inflammatory and gut microbiota regulatory effects^14^. In addition, our previous study found that the combined use of PC-01 and B8589 in milk fermentation significantly increased the levels of GABA and short-chain fatty acids in the produced fermented milk compared with using the PC-01 strain alone, which likely enhances its probiotic function^13^. Therefore, this study aimed to further investigate the milk metabolomic changes during the co-fermentation and fermented milk ripening processes when both strains were used together as the dairy starter. Changes in the viable counts and milk metabolomics were monitored by flow cytometer and nontargeted metabolomics, respectively (Fig. 1).

## Results

### Changes in the viable cell count in the probiotic fermented milk

**Fig. 2:**
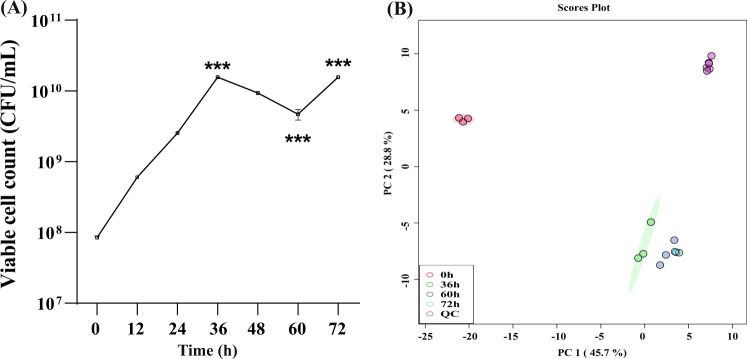
Changes in the probiotic viability and milk metabolomes of the fermented milk. **A** Changes in the viable cell count in the probiotic fermented milk. Error bars represent mean ± SD. ***P < 0.001. Differences in microbial viable counts between time points were evaluated using ANOVA at the 95% significance level (*P* < 0.05). **B** Principal component analysis score plot of milk metabolomes at different time points, i.e., 0, 36, 60, and 72 h, of fermentation. QC quality control samples.

The viable cell count of the probiotic fermented milk was detected at different time points of fermentation using flow cytometry (Fig. 2A). The viable cell count reached a peak at 36 h of fermentation (1.57 × 10^10^ CFU/mL), which was significantly higher than at 0 h (8.49 × 10^7^ CFU/mL; *P* < 0.001). It decreased significantly to 8.87 × 10^9^ CFU/mL at 60 h (*P* < 0.001) and rose to 1.57 × 10^10^ CFU/mL at 72 h (*P* < 0.001).

### Metabolic footprint of compound probiotic fermented milk at different fermentation stages

Untargeted metabolomics (based on UPLC-QE-MS) was performed on the probiotic fermented milk samples collected at 0, 36, 60, and 72 h. To improve the interpretability and to validate our data, principal component analysis and orthogonal partial least squares-discriminant analysis (OPLS-DA) were performed to visualize changes in the milk metabolome at different time points. Principal component analysis is an unsupervised machine learning algorithm that reflects the overall variability between samples. The *x* and *y* axis, respectively, show the projected scores of samples on the plane composed of the first and second principal components, which can provide reference for the similarity or difference between samples. Symbols representing samples of 0, 36, 60, and 72 h showed distinct time-based clustering trend, and the quality control samples also clustered closely as a group (Fig. 2B), suggesting that the overall structure of the milk metabolome varied between time points, and that the equipment and chromatography conditions were stable and reliable.

**Fig. 3:**
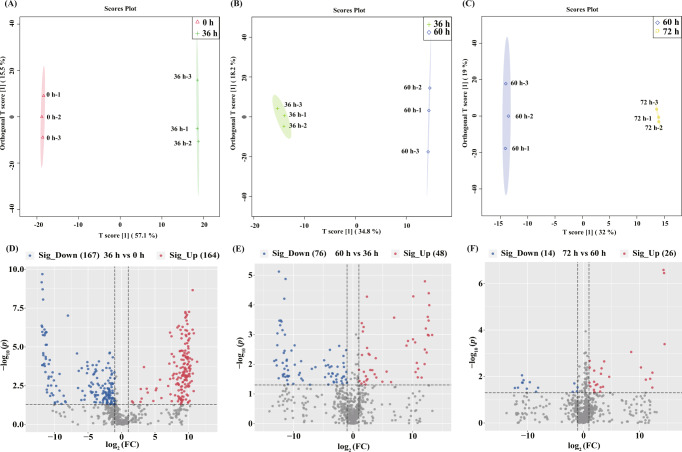
Time-course changes in the metabolomic profile of probiotic fermented milk during fermentation. **A**–**C** Orthogonal partial least squares-discriminant analysis score plots and **D**–**F** volcano plots of fermented milk samples. **A**, **D** 0 h versus 36 h; **B**, **E** 36 h versus 60 h; **C**, **F** 60 h versus 72 h. The volcano plots illustrate the milk metabolomics data. Each dot represents a detected metabolite. Significantly increased (Sig_Up) and decreased (Sig_Down) metabolites found by pairwise comparison between samples are shown in red and blue, respectively (cut-off *P* < 0.05 and fold change [FC] >2 or <0.5). The significant thresholds are marked by the black dotted lines in the volcano plots. Differential metabolites between time points were evaluated using ANOVA at the 95% significance level (*P* < 0.05).

Then, OPLS-DA models were used to identify differential marker metabolites between time points (0 h versus 36 h; 36 h versus 60 h; 60 h versus 72 h). The OPLS-DA modeling is based on supervised classification. It filters out the orthogonal variables that are not related to the categorical variables of metabolites and analyzes the non-orthogonal variables and orthogonal variables separately, so as to obtain more reliable differential metabolites between groups. The score plots of OPLS-DA models are shown in Fig. 3A–C. Again, a clear time-based clustering trend is observed in each pairwise comparison. In addition, the model parameters for each group confirmed the validity and accuracy of the models (0 h versus 36 h: R^2^Y = 0.999, Q^2^ = 0.997; 36 h versus 60 h: R^2^Y = 0.992, Q^2^ = 0.983; 60 h versus 72 h: R^2^Y = 0.994, Q^2^ = 0.988).

### Identification of differential marker metabolites

Detected metabolites are visualized in volcano plots (Fig. 3D–F), and the cut-off thresholds for differential marker metabolites were fold change ˃2 or <0.05 and *P* < 0.05. A total of 304 differential metabolites (151 increased and 153 decreased at 36 h compared with 0 h) were identified, 244 of which were identified by database searches (Supplemental Table 1), including 66 lipids and lipid-like molecules, 59 organic acids and derivatives, 43 organic oxygen compounds, 31 organ heterocyclic compounds, 13 phenylpropanoids and polyketides, 15 benzenoids and seventeen other metabolites. One hundred and ten differential metabolites were identified between probiotic milk metabolomes at 36 h and 60 h (43 increased and 67 decreased at 60 h compared with 36 h), and 79 of these differential metabolites were identified by database searches (Supplemental Table 1), including 21 organic oxygen compounds, 20 organic acids and derivatives, 17 organ heterocyclic compounds, nine lipids and lipid-like molecules, and 12 other metabolites. Thirty-six differential metabolites were identified between 60 h and 72 h (24 increased and 12 decreased at 72 h compared with 60 h), and 27 of these differential metabolites were identified by database searches (Supplemental Table 1), including seven lipids and lipid-like molecules, nine organic oxygen compounds, five organic acids and derivatives, and six other metabolites.

**Fig. 4:**
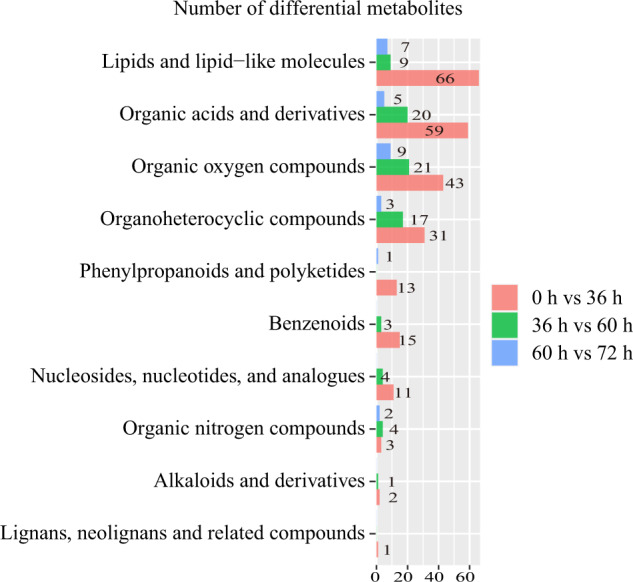
Types of differential metabolites identified between probiotic fermented milk samples collected at different time points. The pink, green, and blue horizontal bars represent the number of differential metabolites (written next to/on the bars) identified between 0 h and 36 h; 36 h and 60 h; 60 h and 72 h, respectively.

We then ranked the key differential metabolites by chemical nature to gain further understanding of the biochemical process in probiotic fermented milk production (Fig. 4). The four main types of differential metabolites belonged to lipids and lipid-like molecules, organic acids and derivatives, organic oxygen compounds, and organ heterocyclic compounds, followed by phenylpropanoids and polyketides, benzenoids, alkaloids and derivatives and so on. Obviously, different differential metabolic profiles (both in metabolite diversity and abundance) were found between time points (0 h versus 36 h; 36 h versus 60 h; and 60 h versus 72 h). To further investigate biochemical changes at different stages of milk fermentation, we selectively performed metabolic pathway enrichment analysis on three major types of metabolites (i.e., lipids and lipid-like molecules, organic acids and their derivatives, organic oxygen compounds).

### Metabolic pathway enrichment analysis

**Fig. 5:**
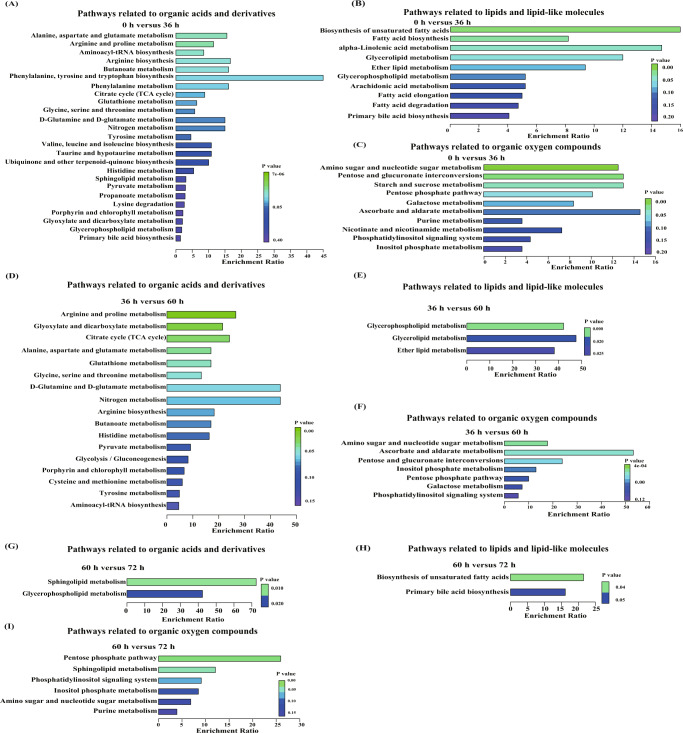
Enrichment analysis of differential metabolic pathways identified between different fermentation time points. Comparison between (**A**–**C**) 0 h and 36 h; **D**–**F** 36 h and 60 h; and **G**–**I** 60 h and 72 h. The enriched pathways are related to organic acids and derivatives (**A**, **D**, **G**), lipids and lipid-like molecules (**B**, **E**, **H**), and organic oxygen compounds (**C**, **F**, **I**), respectively. The color scale represents the confidence of significant difference (*P* value) between pairs of samples. Differential metabolic pathways were detected by the Wilcoxon rank-sum test.

Metabolite pathway enrichment analysis is an effective tool to elucidate the mechanisms of metabolic alterations of the samples. From 0 to 36 h, 25 organic acids and derivatives-related metabolic pathways were identified, involving 17 different metabolites (mainly amino acids and organic acids; Fig. 5A and Supplemental Table 2). Ten lipids and lipid-like molecules-related metabolic pathways were enriched, involving nine different metabolites (mainly fatty acids and glycerophospholipids; Fig. 5B and Supplemental Table 2). Ten organic oxygen compounds-related metabolic pathways were identified, involving 13 different metabolites (mainly carbohydrates; Fig. 5C and Supplemental Table 2).

From 36 to 60 h, 17 organic acids and derivatives-related metabolic pathways were identified, involving 6 different (mainly amino acids and organic acids; Fig. 5D and Supplemental Table 2). Three lipids and lipid-like molecules-related metabolic pathways were enriched, involving two different glycerophospholipids metabolites (Fig. 5E and Supplemental Table 2). Seven organic oxygen compounds-related metabolic pathways were identified, involving seven different metabolites (mainly carbohydrates; Fig. 5F and Supplemental Table 2).

From 60 to 72 h, two organic acids and derivatives-related metabolic pathways were identified, involving one organic acid (Fig. 5G and Supplemental Table 2). Two lipids and lipid-like molecules-related metabolic pathways were enriched, involving, involving two different metabolites (mainly fatty acids and bile acids; Fig. 5H and Supplemental Table 2). Six organic oxygen compounds-related metabolic pathways were identified, involving five different metabolites (mainly sugars, carbonyl compounds and alcohols**;** Fig. 5I and Supplemental Table 2).

## Discussion

**Fig. 6:**
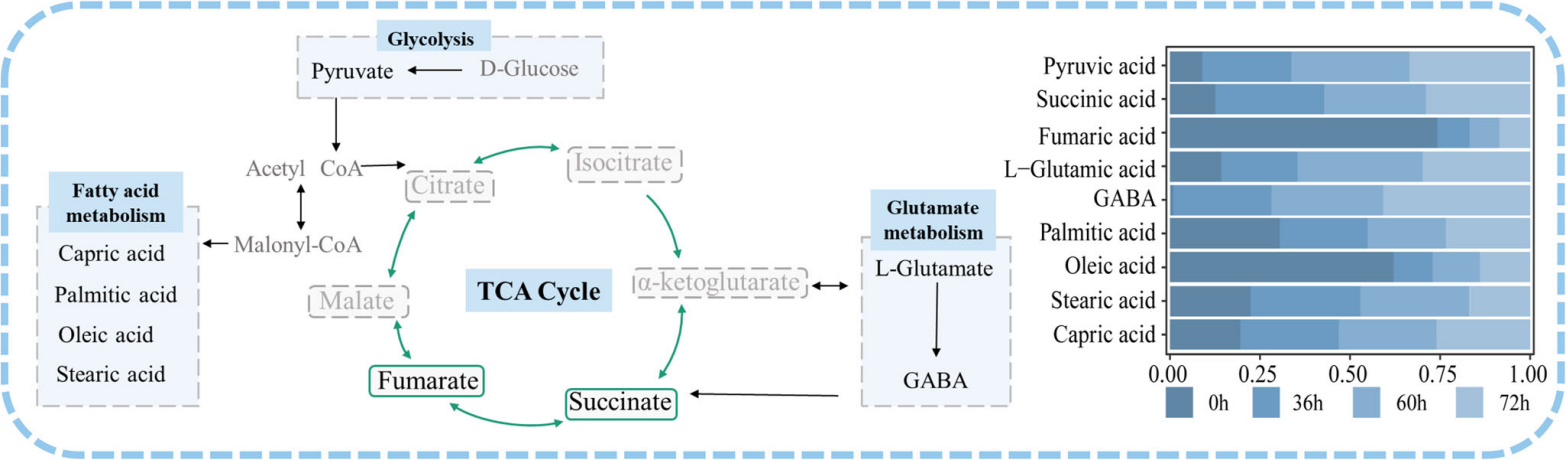
Metabolic map constructed from nine identified differential metabolites and their relative abundances. The nine metabolites (namely pyruvic acid, succinic acid, fumaric acid, l-glutamic acid, GABA, palmitic acid, oleic acid, stearic acid, and capric acid) are written in black in the metabolic map (left panel). The horizontal bar chart (right panel) shows that the relative abundance of these metabolites at different sample collection time points, i.e., 0, 36, 60, and 72 h. CoA co-enzyme A, GABA γ-aminobutyric acid, TCA tricarboxylic acid.

This study used LC-MS-based metabolomics to investigate the metabolic changes in the probiotic milk metabolome at different time points during complex fermentation by the bacterial strains, PC-01 and B8589. By comparing metabolites at different time points, some significant differential biomarkers have been identified. These metabolites are mainly organic acids, amino acids, and fatty acids, which are involved in important metabolic pathways like the tricarboxylic acid (TCA) cycle, fatty acid metabolism, and glutamate metabolism (Fig. 6). These metabolites and the metabolic pathways they participate in not only affect the growth and reproduction of the probiotic bacteria in the milk environment, but also influence the flavor and probiotic properties of the fermented milk. Metabolic pathways are complex, and each pathway can promote or inhibit each other via feedback mechanism of the produced metabolites. To the best of our knowledge, the interactions of some of the currently found metabolites and their associated metabolic pathways/specific effects in milk fermentation have not been reported previously, which merit further investigation. Nine fermentation stage-specific metabolites (succinic acid, pyruvic acid, l-glutamic acid, fumaric acid, GABA, capric acid, oleic acid, palmitic acid, stearic acid) significantly contributed to differentially enriched metabolism (Fig. 6). These metabolites are ubiquitous in fermented milk and are important for its quality and sensory properties.

Most of the differential metabolites are organic acids. The most common organic acids are carboxylic acids (R-COOH), and their acidity comes from the carboxyl group (−COOH). Short-chain carboxylic acids are important chemical factors affecting the flavor of dairy products. These acids originate from lipolysis, carbohydrate metabolism, and amino acid metabolism^15^. Biomarker short-chain carboxylic acids found in this study include succinic acid, pyruvic acid, and fumaric acid, which are generated in the metabolism of lactic acid bacteria^7,16,17^.

Pyruvate is formed by decomposing glucose 6-phosphate formed after glucose phosphorylation by hexokinase^18^. It is the final product of glycolysis, which gives yogurt an acetic acid aroma and a pleasant sour taste. This study found a significant increase in the pyruvate from 0 to 36 h, accompanied by the rapid growth of probiotics during this fermentation stage. The rapid growth of bacteria is likely due to the initially rich glucose environment and a higher pyruvate anabolism than catabolism. As fermentation ends, glucose is depleted, and more pyruvate is converted to other downstream metabolites, slowing bacterial growth. Pyruvate is also a precursor for acetyl-CoA, an important substrate of the TCA cycle^15^. Succinic acid and fumaric acid are intermediates of the TCA cycle. Succinic acid, with its sour, salty, and bitter tastes, is one of the mitochondrial signaling molecules that regulates oxidative stress and inflammatory responses in cells^19^. Fumaric acid is the simplest unsaturated dicarboxylic acid in nature with a fruity sour taste^20^. Succinic acid can be oxidized to fumaric acid by succinate dehydrogenase^19^. Similar to pyruvate, this study found a significant increase in succinic acid at 36 h. However, the content of fumaric acid continued to decrease and did not increase with the accumulation of succinic acid. This may implicate that the probiotic bacteria consumed fumaric acid. Some lactic acid bacteria (such as *Limosilactobacillus reuteri* and *Lacticaseibacillus casei*) can utilize the TCA cycle in the direction of the reduction reaction, i.e., reducing fumaric acid to form succinic acid to promote bacterial growth^21^. In addition, fumarate does not accumulate in excess during normal metabolism, and it is quickly catalyzed by fumarate hydratase to generate malate^22^. We indeed detected D-malic acid as a differential metabolite, although it did not significantly increase at 36 h compared with 0 h. This may be another reason for the continuous decline in fumaric acid.

The metabolism of amino acids is closely related to the TCA cycle, and amino acids are also important products of nitrogen source metabolism. l-glutamate and α-ketoglutarate connect amino acid metabolism and TCA cycle through the GABA metabolic pathway^23^. The metabolic pathway of GABA is a branch derived from the TCA cycle, which is a process in which microorganisms directly and irreversibly decarboxylate l-glutamate through glutamate decarboxylase to synthesize GABA^24^. GABA is a four-carbon non-protein amino acid, which has various functions, such as lowering blood pressure, anticonvulsant effect, calming nerves, improving liver and kidney, strengthening the body’s antioxidant capacity, increasing exercise tolerance, enhancing immune function and reproductive capacity^25^. One fermentative pathway is the microbial conversion of glutamate to GABA, which is triggered by the decreased pH during fermentation^26^. Our results showed a continuous increase in the GABA content, which peaked at the end of the fermentation, correlating to fermented milk acidification. At the same time, GABA is also a growth factor for some bacteria, which has been reported to promote the growth of probiotics in some fermentation systems^24^. This is in line with the current observation of an active bacterial growth with a high viability of probiotics. On the other hand, glutamate imparts a salty taste at high concentrations^27^, and its conversion to GABA through fermentation improves the sensory quality of the probiotic fermented milk.

Fatty acids are usually produced by chemical processes such as lipolysis, protein hydrolysis, and lactose fermentation; and fatty acid metabolism links to the TCA cycle via the substrate, acetyl-CoA^11^. Fatty acids not only improve the taste, flavor, and texture of the product, but also have a physiological impact on the consumers. The main unsaturated fatty acid is oleic acid, which accounts for 70% of the total unsaturated fatty acids in fermented milk^28^. Oleic acid can regulate blood lipids, lower cholesterol, and improve insulin sensitivity; it can also reduce oxidative stress and excessive inflammatory responses in critically ill patients^29^. Several studies detected decreases in oleic acid in dairy products during fermentation^11,30^, which is consistent with our results. Our study found that the content of oleic acid decreased significantly after 36 h of fermentation, which may be explained by the conversion of oleic acid to stearic acid through hydrogenation^11^. In fact, we observed an increase in stearic acid at 36 h. Epidemiological studies have found that ingesting various fatty acids have different biological consequences^31^. Increased levels of stearic acid have been associated with lowering of blood pressure, improvement in heart function, and reduction in cancer risk^32^. Contrary to the popular belief that saturated fatty acids are harmful, stearic acid appears to have some beneficial effects on human health, although the molecular mechanism of this phenomenon is still unclear^31,33^. Another common fatty acid detected in fermented milk is palmitic acid, which is generally considered unhealthy^34^. However, most studies only analyzed the effect of a high dose of palmitic acid^32,35^, and only one study has demonstrated desirable apoptotic and anti-cancerous effects of palmitic acid in probiotic fermented milk^36^. Although dairy products are an important dietary source of palmitic and stearic acids, their intake have been associated with a lower risk of cardiometabolic disease^37^. Thus, the increases in palmitic and stearic acids through milk fermentation could be desirable, but more studies will be needed to confirm the health effects of these metabolites. Our results showed that the level of capric acid notably increased at 36 h of fermentation and remained at a high level until the end of fermentation. Capric acid is a medium-chain fatty acid found in some fermented milks that improves epilepsy, boosts brain energy metabolism, and alleviates nutrient malabsorption syndrome^38^.

In conclusion, this study generated snapshots of bacterial viability and probiotic fermented milk metabolomes at different time points during and after milk fermentation using UPLC-QE-MS. The probiotic fermented milk metabolome changed greatly at earlier time points (between 0 and 36 h), and showed only mild differences during the interim period (between 36 and 60 h) and ripening (between 60 and 72 h). A number of differential metabolites were identified between different stages of probiotic milk fermentation, mainly including organic acids, amino acids and fatty acids, involving in the TCA cycle, glutamate metabolism, and fatty acid metabolism. Nine differential metabolites were identified as time point-specific differential biomarkers, and their changes may lead to the unique sensory and nutritional quality of probiotic fermented milk. Some functional biomolecules, e.g., pyruvate, GABA, and capric acid, increased significantly at the end of fermentation, which might contribute to the beneficial properties of probiotic fermented milk. This time-course metabolomics study analyzed probiotic-specific fermentative changes in milk, providing detailed information of probiotic metabolism in a milk matrix.

## Methods

### Fermented milk production

Skim milk powder (NZMP, Wellington, New Zealand) with 2% glucose was dissolved in distilled water (50 °C, 30 min). The milk was homogenized (65 °C, 20 MPa) and pasteurized (95 °C, 5 min) before cooling to 20 °C. The bacterial strains, B8589 and PC-01, were used for complex fermentation. Both strains were probiotics provided from the Lactic Acid Bacteria Culture Collection, Inner Mongolia Agricultural University, China. The probiotics were inoculated to the cooled pasteurized skim milk, and their initial viable counts were adjusted to 1 × 10^7^ CFU/mL for PC-01 and 1 × 10^6^ CFU/mL for B8589, respectively. The inoculated milk was anaerobically fermented at 37 °C until the pH reached 3.8 after 72 h. After reaching pH 3.8, the probiotic fermented milk was stored at −80 °C until further analysis. Fermented milk samples were collected every 12 h during the fermentation process. The study design is illustrated in Fig. 1.

### Enumeration of viable probiotic bacteria by flow cytometer

The viable count of probiotic bacteria in the fermented milk was enumerated as previously described^39^. Two stains, SYTO 9 (5 mM) and propidium iodide (PI; 1 mg/ml), were respectively dissolved in DMSO ( ≤1%). The fermented milk samples were diluted to the density of 10^6^ to 10^7^ CFU/mL, and 980 μL of diluted fermented milk samples were transferred to four clean tubes. In three of the tubes, 10 μL PI (0.2 mM/L; PI single staining), 10 μL SYTO 9 (0.1 mM/L; SYTO 9 single staining), and 10 μL of mixed stains (SYTO 9, 0.1 mM/ L; PI 0.2 mM/L; double staining) were added, respectively. All four centrifuge tubes were shaken for 30 s and incubated at room temperature in the dark for 15 min. Aliquots of double-stained samples (200 μL each) were mixed with an equal amount of absolute counting microspheres (Flow Counting Fluorescent Spheres; Beckman Coulter Inc., Brea, CA, USA) for analysis on a flow cytometer (MoFlo Astrios EQ Cell Sorter; Beckman Coulter Inc., Brea, CA, USA).

The light source of the flow cytometer was an air-cooled argon-ion laser (excitation light wavelength at 488 nm; emission light wavelength >630 nm). For each sample, data from 500,000 cells were collected. Unstained samples and PI fluorescently stained bacterial samples were used as controls. Forward scatter-side scatter dot plots were drawn, and R1 gate was set to delineate target cells. A scatter plot with 488-513/26-Height-Log as the abscissa and 561-614/20-Height-Log as the ordinate was established to delineate the number of SYTO 9 positive and PI negative bacteria. A scatter plot with 488-710/45-Height-Log as the horizontal coordinate and 488-SSC-Area as the vertical coordinate was set up to enumerate absolute count microspheres. Total bacterial count = viable bacterial count/absolute count of microspheres × absolute count of microspheres concentration × dilution ratio.

### Sample preparation for untargeted metabolomic analysis

Samples (100 μL each) were mixed with 400 μL of extraction solution (acetonitrile: methanol = 1:1, containing isotopically labeled internal standard mixture) for 30 s in Eppendorf tubes. The mixtures were sonicated for 10 min on ice water, left to stand at −4 °C for 1 h, and centrifuged at 4 °C, 12,000 rpm (centrifugal force 13,800 × *g*, radius 8.6 cm) for 15 min. The sample supernatants were filtered and transferred to clean sample vials for ultra-performance liquid chromatography analysis (UPLC) with an Acquity UPLC (Vanquish, Thermo Fisher Scientific Inc., Waltham, MA, USA) coupled to Q Exactive HF-X Hybrid Quadrupole-Orbitrap Mass Spectrometer (Thermo Fisher Scientific Inc., Waltham, MA, USA). An Acquity UPLC BEH Amide column (2.1 mm × 100 mm, 1.7 μm) was used for analyte separation (Waters Corporation, Milford, MA, USA). An equal amount of all samples was mixed to composite the QC sample to be run to ensure the stability of the instrumental and chromatographic conditions. The liquid chromatography phases were: an aqueous phase A, containing 25 mmol/L ammonium acetate and 25 mmol/L ammonia water; phase B was acetonitrile. The gradient elution was programmed as follows: 0–0.5 min, 95% B; 0.5–7 min, 95–65% B; 7–8 min, 65–40% B; 8–9 min, 40% B; 9–9.1 min, 40–95% B; 9.1–12 min, 95% B. The sample pan temperature was 4 °C, and the injection volume was 2 μL. The QE HFX mass spectrometer was used for its ability to acquire MS/MS spectra on information-dependent acquisition (IDA) mode in the control of the acquisition software (Xcalibur, Thermo). In this mode, the acquisition software continuously evaluates the full-scan MS spectrum. The electrospray ionization source conditions were set as follows: sheath gas flow rate of 3.35 L/min; aux gas flow rate of 16.8 L/min; the capillary temperature of 350 °C; mass range of 70–1050; Scan/cycle time of 760 ms; full MS resolution of 60,000; MS/MS resolution of 7500; collision energy of 10/30/60 in normalized collision energy mode; spray voltage of 3.6 kV (positive ion mode) or 3.2 kV (negative ion mode), respectively.

The original data file was converted by ProteoWizard software to mzXML format. A self-written R package (XCMS kernel) was used to process data for peak identification, extraction, alignment and integration, and an in-house MS2 database (BiotreeDB) was used for metabolite annotation.

### Statistical analysis

Differences in microbial viable counts between time points were evaluated using ANOVA at the 95% significance level (*P* < 0.05) by using R version 4.0.4 (R Foundation for Statistical Computing, Vienna, Austria). Metabolomic changes between time points were visualized using principal component analysis and OPLS-DA, which were constructed by the online tool, Metaboanalyst 5.0 (https://www.metaboanalyst.ca). Detected metabolites were visualized by volcano plots, and significantly differential metabolic features were selected according to fold change (fold change >2 or <0.5) and *P* value (*P* < 0.05). Metabolic pathways annotation and enrichment analysis of differential metabolites were performed using the Kyoto Encyclopedia of Genes and Genomes database (http://www.genome.jp/kegg/pathway.html). A schematic diagram of the study design was constructed by the online tool, BioRender (https://app.biorender.com/).

### Reporting summary

Further information on research design is available in the Nature Research Reporting Summary linked to this article.

## Supplementary information

Supplemental Table 1

Supplemental Table 2

Reporting Summary

## Data Availability

We declare that all data related to this study are included in this paper and its supplementary information.
